# Structural Analysis of the Interactions Between Paxillin LD Motifs and α-Parvin

**DOI:** 10.1016/j.str.2008.08.007

**Published:** 2008-10-08

**Authors:** Sonja Lorenz, Ioannis Vakonakis, Edward D. Lowe, Iain D. Campbell, Martin E.M. Noble, Maria K. Hoellerer

**Affiliations:** 1 Laboratory of Molecular Biophysics, University of Oxford, Oxford OX1 3QU, United Kingdom; 2 Department of Biochemistry, University of Oxford, Oxford OX1 3QU, United Kingdom

**Keywords:** PROTEINS

## Abstract

The adaptor protein paxillin contains five conserved leucine-rich (LD) motifs that interact with a variety of focal adhesion proteins, such as α-parvin. Here, we report the first crystal structure of the C-terminal calponin homology domain (CH_C_) of α-parvin at 1.05 Å resolution and show that it is able to bind all the LD motifs, with some selectivity for LD1, LD2, and LD4. Cocrystal structures with these LD motifs reveal the molecular details of their interactions with a common binding site on α-parvin-CH_C_, which is located at the rim of the canonical fold and includes part of the inter-CH domain linker. Surprisingly, this binding site can accommodate LD motifs in two antiparallel orientations. Taken together, these results reveal an unusual degree of binding degeneracy in the paxillin/α-parvin system that may facilitate the assembly of dynamic signaling complexes in the cell.

## Introduction

Cell adhesion and migration are coordinated by dynamic membrane-associated protein assemblies called focal adhesions (FAs). With over 150 components, FAs play a crucial role in transmitting signals bidirectionally across the cell membrane and provide a physical link between integrin receptors and the actin cytoskeleton (Zaidel-Bar et al., 2007). The ordered recruitment of signaling and cytoskeletal proteins to FAs relies on a number of adaptor proteins, such as paxillin. Paxillin is composed of a 33 kDa N-terminal region and four C-terminal LIM domains. It is among the most-connected FA proteins and is implicated in the localization of various proteins during adhesion assembly (Brown and Turner, 2004). Many of its ligands bind to highly conserved leucine-rich sequences with the consensus LDXLLXXL (“LD motifs”), located in the N-terminal region of paxillin. LD-binding proteins include the kinases FAK (Turner et al., 1999), ILK (Nikolopoulos and Turner, 2001), and PAK3 (Hashimoto et al., 2001), the Arf-GAP PKL (Turner et al., 1999), the antiapoptotic protein Bcl-2 (Sheibani et al., 2008), the papillomavirus oncoprotein E6 (Tong et al., 1997), and the cytoskeletal proteins vinculin (Turner et al., 1999) and α-parvin (Nikolopoulos and Turner, 2000). While several of these proteins, such as FAK, PKL, and vinculin, bind LD motifs through parallel α-helical bundles (Gao et al., 2004; Hoellerer et al., 2003; Liu et al., 2002; Scheswohl et al., 2008; Schmalzigaug et al., 2007; Zhang et al., 2008), others employ topologically distinct modules, such as the kinase domain of ILK (Tu et al., 2001), the BH4 domain of Bcl-2 (Sorenson, 2004), and the calponin homology (CH) domain of α-parvin (Nikolopoulos and Turner, 2000).

α-parvin (Olski et al., 2001), also known as actopaxin (Nikolopoulos and Turner, 2000) or CH-ILKBP (Tu et al., 2001), is part of the ILK signaling complex (Tu et al., 2001), plays an essential role in adhesion-dependent PKB/Act activation (Fukuda et al., 2003) and in the regulation of actin organization (LaLonde et al., 2005) and Rac activation (LaLonde et al., 2005; Zhang et al., 2004). It belongs to the highly conserved parvin family that shares a common architecture composed of a variable N-terminal region followed by two CH domains (Olski et al., 2001). CH domains are found in a variety of cytoskeletal and signaling proteins, including calponin, spectrin, plectin, fimbrin, and α-actinin, and often occur in tandem to form actin-binding domains (ABDs). However, the primary sequences of both CH domains of α-parvin are highly diverged from the typical type-1 and type-2 CH domains found in ABDs; they have therefore been classified as type-4 and type-5 CH domains (Gimona et al., 2002). An ability to recognize paxillin LD motifs has only been reported for the type-5 CH domains (Nikolopoulos and Turner, 2000; Yoshimi et al., 2006).

Here, we elucidate the molecular basis for selective LD recognition by type-5 CH domains. While this manuscript was in preparation, an independent study reported the NMR structure of the C-terminal CH domain of α-parvin in complex with a 10-residue peptide derived from the paxillin LD1 motif (Wang et al., 2008). Our study significantly extends those findings and provides a comprehensive description of LD recognition by α-parvin, revealing a surprising degree of promiscuity in LD mediated interactions.

We present the first high-resolution crystal structure of α-parvin-CH_C_ and show that it interacts with all five paxillin LD motifs in solution. Three cocrystal structures of α-parvin-CH_C_ with 20-residue peptides representing LD1, LD2, and LD4 allow us to characterize the interaction at atomic resolution and to highlight binding-induced conformational changes in α-parvin-CH_C_. This analysis together with NMR studies of a spin-labeled LD1 peptide supports the surprising finding that LD motifs can associate with a single binding site bidirectionally. Using the full-length protein we further demonstrate that the N-terminal region of α-parvin is not involved in LD-recognition, providing further validation for our molecular model of LD recognition by α-parvin-CH_C_.

## Results

### Crystal Structure of the C-Terminal Type-5 CH Domain of α-Parvin

**Figure 1:**
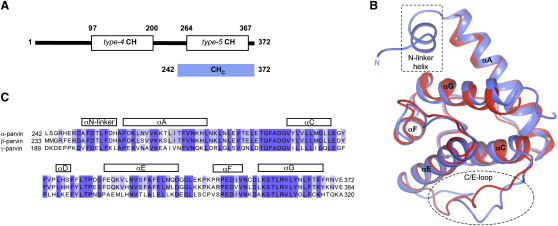
Structure of α-Parvin-CH_C_ (A) Schematic of the domain structure of α-parvin according to SMART (Schultz et al., 1998) and the crystallized fragment α-parvin-CH_C_. (B) Superposition of the ribbon representations of α-parvin-CH_C_ in blue and the type-1 CH domain of α-actinin 3 (residues 42 to 149 of 1WKU) in red. Canonical secondary structural elements include helices αA (258–279), αC (294–304), αE (320–336), αF (346–350), and αG (354–368). Features unique to α-parvin, such as the N-linker helix (249–256) and the C/E-loop containing the 3-residue insertion (313–315), are highlighted. (C) Sequence alignment of α-parvin-CH_C_ with type-5 CH domains of other members of the parvin family. Secondary structure elements are indicated as above. Note that αD (310–312) is only found in 1 of 2 molecules in the asymmetric unit.

We initially attempted to solve the crystal structure of full-length human α-parvin, but no crystals could be obtained. However, limited proteolysis led to the identification of a stable fragment, α-parvin-CH_C_, that readily crystallized. This fragment includes residues 242–372 and thereby spans the entire C-terminal CH domain as defined by SMART (Schultz et al., 1998) and a portion of the inter-CH domain linker (Figure 1A). The crystal structure of α-parvin-CH_C_ at 1.05 Å resolution was determined by molecular replacement using an ensemble of homologous type-1 CH domain structures as search model. Data collection and refinement statistics are summarized in Table 1. The refined structural model includes α-parvin residues 246–372 (molecule A) or 247–372 (molecule B) and represents the first high-resolution crystal structure of a type-5 CH domain (Figure 1B). In spite of very low levels of sequence conservation (≤26% identity) compared to canonical type-1 CH domains (see Figure S1 available online), α-parvin-CH_C_ exhibits a typical CH domain core composed of four main α-helices (αA, αC, αE, and αG; nomenclature from Djinovic Carugo et al., 1997), which are connected by loops and shorter helical elements (αD and αF). Secondary structure matching (Krissinel and Henrick, 2004) of α-parvin-CH_C_ with its closest homolog, the type-1 CH domain of α-actinin 3 (PDB: 1WKU), yields an RMSD of 1.19 Å in 103 equivalent C^α^-positions. Atypically, however, the N-terminal boundary of α-parvin-CH_C_ is extended by a short α-helix comprising residues 249 to 256. This “N-linker helix” tightly associates with helices A and G of the canonical CH domain through electrostatic interactions (of residues D248, D251, and D255 with K355 and R359 in αG) and hydrophobic contacts (of residues F250, L253, and F254 with L354, K355, L358, and R359 of αG and K260 and L261 of αA). We therefore conclude that the N-linker helix forms an integral part of the type-5 CH domain of α-parvin, which is in agreement with its resistance to proteolysis. Another feature peculiar to this type-5 CH domain is the loop between helices C and E, which contains a 3 (or 4)-residue insertion (relative to type-1 CH domains) that is conserved throughout the parvin family (Figures 1C and S1). However, conformational differences in this region may not be significant, since the C/E-loop structure varies between α-parvin-CH_C_ molecules both in the same and different crystal forms and is involved in crystal packing (data not shown).

### Interaction of α−Parvin-CH_C_ with Paxillin LD Peptides in Solution

**Figure 2:**
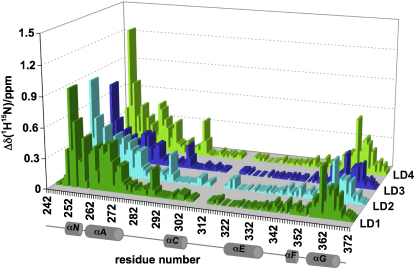
NMR Titrations of α-Parvin-CH_C_ with Paxillin LD Peptides Weighted combined chemical shift perturbations extrapolated to saturating ligand concentrations along the sequence of α-parvin-CH_C_. Grey patches denote unassigned residues or residues broadened due to intermediate exchange (residues 253 and 268). Note that the overall higher amplitudes for LD4 might be due to the relatively high error in the extrapolation to full saturation. LD5 was excluded from this plot, since full saturation could not be achieved.

On the basis of primary sequence comparison with other LD-binding proteins, such as FAK and vinculin, and mutation studies, the LD-binding site (or “paxillin binding subdomain” [PBS]) of α-parvin was mapped to residues 274–291 (Nikolopoulos and Turner, 2000) (i.e., the A/C-loop of α-parvin-CH_C_) (Figure 1B). However, we previously demonstrated that LD-binding to the FAT domain of FAK does not reside in a local peptide sequence such as the PBS (Hoellerer et al., 2003) and thus investigated the interaction of α-parvin-CH_C_ with LD motifs using solution NMR. ^1^H-^15^N HSQC monitored titrations of ^15^N-enriched α-parvin-CH_C_ were performed with peptides representing all five paxillin LD motifs. Each peptide was found to induce resonance-specific chemical shift perturbations (Figure 2 and data not shown), indicating an interaction with α-parvin-CH_C_. Global fitting of the resulting binding curves shows that the affinities of individual LD motifs for α−parvin-CH_C_ differ substantially (Table 2; Figure S2) with LD1 being the highest affinity ligand followed by LD4 and LD2. All three bind with affinities in the micromolar range, while LD3 and LD5 bind in the millimolar range. The overall pattern of chemical shift perturbations induced by saturating concentrations of different LD peptides is very similar (Figure 2), suggesting that all five LD motifs interact with α-parvin-CH_C_ in a similar fashion. Large chemical shift perturbations (δΔ^1^H^15^N) ≥ 0.2 ppm) map to the N-linker helix, helix A, and helix G, whereas the central region is less affected.

### Crystal Structures of α-Parvin-CH_C_ in Complex with Paxillin LD Motifs

**Figure 3:**
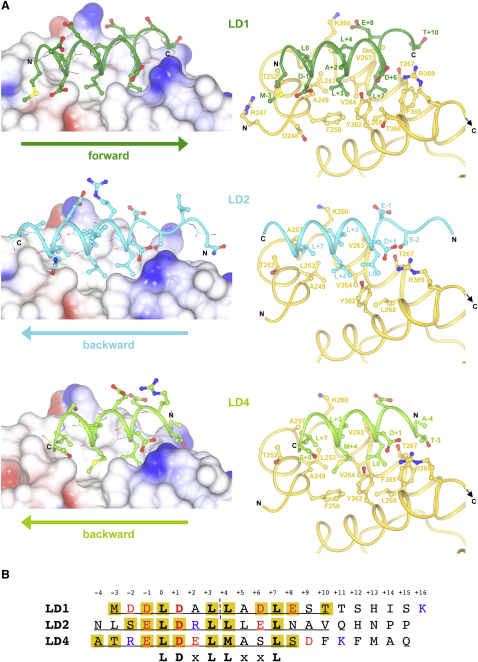
Cocrystal Structures of α-Parvin-CH_C_ with Paxillin LD1, LD2, and LD4 (A) Detail of the α-parvin-CH_C_ complexes with LD1 (top), LD2 (middle), and LD4 (bottom). The left panel shows electrostatic surface renditions of α-parvin-CH_C_ with the bound LD peptides represented by a combination of ribbon, ball-and-stick (side-chains), and cylinder (main chain) modes to indicate directionality. The right panel shows ribbon representations of α-parvin-CH_C_ (gold) and LD peptides including those side-chains within a contact radius of 4 Å as ball-and-stick models. (B) Sequence alignment of LD peptides. Those residues ordered in the crystals are underlined; acidic residues are colored red, and basic ones are blue. Residues in contact with the protein within a radius of 4 Å are boxed. The pseudo-palindromic axis of LD1 is shown as a dashed line.

**Figure 4:**
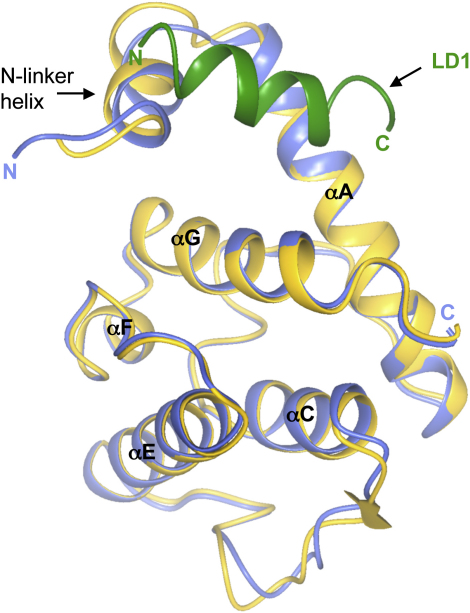
Cocrystal Structure of α-Parvin-CH_C_ with Paxillin LD1 Superposition of the ribbon representations of α-parvin-CH_C_ in blue and its complex with the LD1 peptide in gold and green, respectively. Secondary structural elements are indicated.

To elucidate the molecular basis for LD recognition by α-parvin-CH_C_, we cocrystallized α-parvin-CH_C_ with peptides representing the high-affinity ligands LD1, LD2, and LD4. The corresponding structures at 2.1 Å, 1.8 Å, and 2.2 Å resolution, respectively, were solved by molecular replacement with the α-parvin-CH_C_ *apo* structure (Table 1). In all three cases, continuous positive difference density was identified into which the peptide ligands could be placed unambiguously (Figure S3). The refined structures include residues 247–372 of α-parvin and paxillin residues 1–14, 141–155, and 262–274 for LD1, LD2, and LD4, respectively (Figures 3A and 4). The remaining C-terminal residues of the LD peptides appear disordered, presumably because they do not form contacts with α-parvin-CH_C_ (Figure 3B). All three LD motifs bind to the same binding site on α-parvin-CH_C_ formed by the N-linker helix, the N-terminal part of helix A and the C-terminal part of helix G, which is consistent with our results from chemical shift mapping (Figure 2). We thus conclude that the PBS region previously identified on α-parvin is not directly involved in the interaction with LD peptides.

Surprisingly, however, the orientation of LD1 is reversed compared to LD2 and LD4 in the corresponding crystal structures. To confirm this striking result, we built models corresponding to both possible orientations for each LD-peptide complex and subjected them to simulated annealing refinement using PHENIX (Afonine et al., 2007). Inspection of the resulting difference maps unambiguously confirmed that LD1 is oppositely aligned to LD2 and LD4. From this point forward, the orientation of LD1 in the crystalline state will be denoted as “forward,” and the orientation of LD2/LD4 will be denoted as “backward.”

The observation of bidirectionality in this system was unexpected, since all three LD motifs form amphipathic α-helices when bound and thus do not possess C2-rotational symmetry. Despite their antiparallel orientations, however, the binding modes of different LD peptides are very similar (Figure 3A, Figure S4). As the result of a slight rotation around the helical axis of the bound peptides, the character and position of side chains facing α-parvin-CH_C_ is largely preserved, the same hydrophobic pockets are occupied, and a similar amount of surface area (∼500 Å^2^) becomes buried. We adopt a nomenclature in which the first conserved leucine residue of an LD motif is labeled position 0. The side chains of their conserved leucine residues in position 0, +3, +4, and +7 (Figure 3B) interact with a hydrophobic patch on the surface of the CH_C_ domain formed by residues from the N-linker helix (A249, F250, L253, A257), helix A (V263, V264, and L268) and helix G (Y362 and F365) (Figure 3A). In addition, two positively charged residues (K260 and R369) of α-parvin-CH_C_ are in close proximity to negatively charged residues of the bound LD peptides. Note that these electrostatic contacts involve the conserved aspartate D+1 of the LD consensus in the case of LD2 and LD4, whereas D+6 and E+8 are utilized in LD1. Because of the reverse binding orientation D+1 of LD1 faces away from the binding site and toward R247 and H256 in a symmetry-related copy of α-parvin-CH_C_. On the basis of the probability of the solvation free energy gain upon contact formation (Krissinel and Henrick, 2004), the latter interface is probably an artifact of crystal packing.

Consistent with their similar binding modes (Figure S4), all three LD peptides, irrespective of directionality, impose similar conformational changes in the N-terminal region of α-parvin-CH_C_, whereas the protein core remains virtually unperturbed (Figure 4). In particular, residues 248 to 264, which experience conformational change upon complex formation, are similar in all LD complexes with RMSD-values of 0.28 Å (LD1 versus LD2), 0.23 Å (LD1 versus LD4), and 0.15 Å (LD2 versus LD4) in 16 equivalent C^α^−positions. Compared with α-parvin-CH_C_ *apo*, the angle between the N-linker helix and helix A widens by ∼15° and the N-linker helix rotates slightly. Residues 258–260 of helix A, which are α-helical in the unbound conformation, adopt a 3_10_-helix in the complexes. The short loop surrounding P258 between the N-linker helix and helix A reorganizes to accompany this conformational change, as has been seen for proline-containing hinge-regions that delimit conformational changes in other proteins (Figure 4).

### Directionality of LD1 Binding to α−Parvin-CH_C_ in Solution

We next asked the question whether the direction of LD-binding to a single site on α-parvin-CH_C_ is a function of the primary sequence of the ligand. A sequence alignment shows that the LD1 motif has noticeable pseudo-palindromic features with respect to those residues critical for the interaction with α−parvin (Figure 3B), raising the possibility that it might be able to associate with α-parvin-CH_C_ bidirectionally. To test this hypothesis and to explore the role of electrostatic interactions, LD1 peptide variants were synthesized in which residues D+1 and D+6, which are predicted to form orientation-specific electrostatic contacts, were substituted by alanine either individually or in combination (D+1A, D+6A or D+1A/D+6A). As measured by NMR, none of these substitutions substantially altered the binding affinity for α-parvin-CH_C_, indicating that the predicted electrostatic contacts are energetically neutral under the assay conditions (50 mM sodium phosphate and 100 mM NaCl [pH 6.9]) used. Therefore, analysis of LD1 peptide variants did not define the binding mode of LD1 in solution. Likewise, comparison of the chemical shift perturbations imposed by oppositely aligned peptides on backbone amide resonances of α-parvin-CH_C_ (Figure 2) is inconclusive. We note that the peptide side-chains contacting the LD-binding site of α-parvin-CH_C_ are similar in either orientation (Figure S4), as is the conformational rearrangement induced by peptide binding, so that the perturbation patterns of either unidirectional binding mode (or a bidirectional mixture) may be indistinguishable.

The binding orientation of LD1 in solution was therefore studied by paramagnetic relaxation enhancement (PRE) measurements using an N-terminally PROXYL-labeled peptide derivative. Spin labels increase nuclear relaxation rates and consequently lead to a loss of resonance intensity in a distance dependent manner (usually within a radius of about 20 Å). Consequently, the addition of PROXYL-labeled LD1 to ^15^N-enriched α-parvin-CH_C_ was expected to result in distinct PRE effects depending on binding orientation. The K_D_-values of PROXYL-labeled and underivatized LD1 peptide for α-parvin-CH_C_ were found to be the same (data not shown), suggesting that the spin-label does not significantly perturb the interaction.

**Figure 5:**
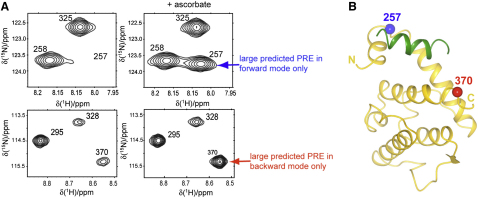
PRE Experiment with Spin-Labeled LD1 (A) Details of the ^1^H-^15^N HSQC spectra of 230 μM ^15^N-enriched α-parvin-CH_C_ and 250 μM PROXYL-labeled LD1 peptide in the absence (left) and presence (right) of 5 mM ascorbate. The latter serves to reduce the spin label, thereby eliminating PRE effects. The binding orientation of LD1 seen in the crystal structure is denoted “forward.” (B) Ribbon representation of α-parvin-CH_C_ in gold and LD1 in green. The position of α-parvin residues 257 and 370 are highlighted in blue and red, respectively.

On the basis of the cocrystal structures of α-parvin-CH_C_ with LD1 and LD4, we calculated theoretical PRE values for all protein NH resonances corresponding to either forward or backward binding modes, respectively. Interestingly, the experimentally derived PRE data resemble a mixture of the two simulated PRE profiles (Figure S5A). This phenomenon is also illustrated in Figure 5, using two diagnostic NMR signals as examples: the resonance originating from residue 257 is predicted to experience strong PRE in the forward mode (calculated distance from spin-label 10.7 Å) but should only be weakly affected in the backward mode (calculated distance 20.2 Å). The opposite behavior should apply to the resonance assigned to residue 370 (calculated distances of 23.3 Å in forward versus 14.1 Å in backward mode). However, our experimental data show that both resonances undergo significant broadening, suggesting that a simple unidirectional model may be insufficient to describe LD1 binding in solution. To quantify this observation, we calculated the linear correlation coefficients, R, between the experimental and the simulated PRE data for various ratios of forward-to-backward binding in a bidirectional mixture (Figure S5B). The resulting curve is bell shaped, indicating that the experimental data are more consistent with a bidirectional than a unidirectional binding model. The best correlation, with a value of ∼0.8, is generated by a model corresponding to a forward-to-backward ratio of ∼75%/25%. Although the improvement on R upon consideration of a bidirectional model is small, F-test analysis between the unidirectional forward and this optimal bidirectional model shows that the improvement in the later is statistically significant (p < 0.001), and argues for the presence of both binding orientations in solution. The observation that no model returns an R value of 1.0 may reflect both the neglect of peptide flexibility in the PRE simulation, in particular the dynamic process of LD1 helix formation on α-parvin-CH_C_, and/or nonspecific effects of the spin label. In conclusion, our analysis provides support for the hypothesis that both antiparallel binding modes are accessible to the LD1 peptide in solution.

### Comparison of LD1 Binding to α-Parvin-CH_C_ and Full-Length α-Parvin

To establish whether the N-terminal region of α−parvin, which is excluded from our structural analysis, contributes to LD recognition, we compared binding of a fluorophore-labeled LD1 peptide to either α-parvin-CH_C_ or full-length α-parvin by fluorescence anisotropy. Both binding curves could be fitted by a single site model, suggesting that α-parvin contains only one LD-binding site located within the CH_C_ fragment (Figure S6). The resulting K_D_-value for α-parvin-CH_C_ is similar to the corresponding value obtained by NMR, supporting the validity of our results (Table 2). Importantly, the K_D_-values for α-parvin-CH_C_ and full-length α-parvin are the same within error (Table 2), suggesting that the N-terminal region of α-parvin (residues 1–242) makes little net contribution to LD binding. We thus conclude that the crystal structures of the α-parvin-CH_C_/LD complexes along with the solution NMR studies presented here provide a relevant description of LD recognition by α-parvin.

## Discussion

We have applied a combination of high-resolution X-ray crystallography and solution techniques to characterize the recognition of paxillin LD motifs by α-parvin. Our results demonstrate that full-length α-parvin contains a single LD binding site, formed by the N-linker helix, helix A, and helix G of the C-terminal type-5 CH domain. This binding site is consistent with the one described in a recent solution NMR structure of α-parvin-CH_C_ in complex with an LD1 peptide (Wang et al., 2008). Using NMR titrations, we have shown that this binding site interacts with all five paxillin LD motifs, exhibiting a preference for LD1, LD2, and LD4 over the less conserved LD3 and LD5. This could be due to differences in specific contacts and/or helical propensity of the latter two LD motifs. In contrast, previous data based on less-sensitive pull-down experiments suggested that α-parvin binding is limited to LD1 and LD4 (Nikolopoulos and Turner, 2000). In our studies, LD1 binds to α-parvin-CH_C_ most tightly and consistently yields a K_D_-value of ∼100 μM independent of the experimental method employed. The significantly lower value of ∼1.2 mM recently measured (Wang et al., 2008) might be due to the restricted length of the peptide (10 versus 20 residues) used in their study.

The relatively low degree of selectivity for individual LD motifs seen for α-parvin-CH_C_ is reminiscent of the low selectivity reported for other LD-binding proteins. The FAT domain of FAK contains two independent LD binding sites, which bind equally well to LD2 and LD4 in vitro (Hoellerer et al., 2003; Gao et al., 2004), but it also interacts to some degree with the other LD motifs (M.K.H., unpublished results). Recent NMR studies (Zhang et al., 2008) have also demonstrated that the single LD binding site of PKL/GIT1 binds to both LD4 (Turner et al., 1999) and LD2. Taken together, these observations suggest that paxillin LD motifs are promiscuous protein interaction modules. This degeneracy can be explained by the high similarity of those residues of individual LD motifs that form the interacting surface of the amphipathic helix (Figures 3 and S4). A higher degree of specificity may be achieved in the context of full-length paxillin and/or through the interplay of several LD binding partners in vivo.

**Figure 6:**
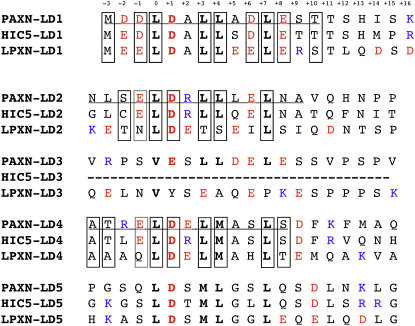
Conservation of α-Parvin Contacting Residues in LD Motifs of Paxillin Paralogs Sequence alignment of LD motifs in human paxillin (PAXN), hic-5 (HIC5), and leupaxin (LPXN). Paxillin residues ordered in the crystal structures of the α-parvin-CH_C_/LD complexes are underlined and those forming contacts with α-parvin-CH_C_ within a radius of 4 Å are boxed.

On the basis of our results for α-parvin, we can make two general predictions with respect to LD recognition by CH domains: First, as noted previously (Wang et al., 2008), we propose that the presence of an N-linker helix is a prerequisite for the interaction with LD motifs. In agreement with this hypothesis, it has been reported that α-actinin, whose canonical CH domains lack this structural element, does not interact with paxillin (Nikolopoulos and Turner, 2000). Since the C-terminal region, including the N-linker helix, is highly conserved throughout the parvin family, we speculate that all parvin paralogs may be able to bind LD motifs. Interestingly, γ-parvin was shown to associate with paxillin in vivo (Yoshimi et al., 2006). Available experiments with β-parvin, however, have proved negative to date (Yamaji et al., 2004), although this protein is more similar in sequence to α-parvin than is γ-parvin (Figure 1C). Second, on the basis of the analysis of differential binding of α-parvin-CH_C_ to individual paxillin-derived LD motifs, we can predict which LD motifs in the paxillin paralogs Hic-5 and leupaxin are likely to be ligands for α-parvin. The sequence alignment in Figure 6 demonstrates that only LD1 is sufficiently conserved in the case of leupaxin, whereas Hic-5 contains three potential α-parvin-binding LD motifs, a hypothesis borne out by experiment (Nikolopoulos and Turner, 2000). Although no interaction of leupaxin with α-parvin has yet been reported, we propose that it will interact with α-parvin via its LD1 motif. Members of the paxillin and parvin families are differentially expressed in human tissues (http://www.hprd.org), and it is possible that they may form tissue-specific complexes.

The structure of α-parvin-CH_C_ is topologically distinct from other LD-binding domains, such as the FAT domain of FAK; it associates with a single LD motif across three oblique helices, whereas FAT accommodates two LD motifs in parallel fashion on opposite sites of its 4-helical bundle (Hoellerer et al., 2003). This suggests that recognition of paxillin LD motifs does not depend on a conserved fold. However, the surface properties of the LD binding sites of α-parvin and FAT are similar, consisting of a hydrophobic patch flanked basic residues. In both cases, LD motifs adopt an α-helical conformation when bound, which allows the conserved leucine residues on one face of the helix to interact with the hydrophobic binding site. Notably, the role of electrostatic contacts in LD recognition might differ between the two proteins: while substitutions of the conserved aspartates (D+1) of LD2 and LD4 to alanine were found to abolish the interaction of N-terminal paxillin fragments with FAT (Brown et al., 1996; Scheswohl et al., 2008; Thomas et al., 1999), our study and others (Wang et al., 2008) show that the equivalent substitutions in the isolated LD1 peptides do not significantly perturb α-parvin-CH_C_ binding in vitro.

Our crystallographic studies reveal that the LD-binding site on α-parvin-CH_C_ can accommodate different paxillin LD motifs in either of two antiparallel orientations. Bidirectional protein-ligand interactions are widespread among PPII (poly-proline type-2) helical ligands, as this conformation has a pseudo C_2_-rotational symmetry perpendicular to its long axis (Ball et al., 2005). In contrast, bidirectional recognition of α-helices is relatively rare, since these ligands do not generally demonstrate C2-rotational symmetry. To our knowledge, this phenomenon has only been reported for the following: the recruitment of the histone deactelylase-associated Sin3 corepressor by the HBP1 and Mad1 repressors, respectively (Brubaker et al., 2000; Spronk et al., 2000; Swanson et al., 2004), the association of FAT with paxillin LD motifs and the endocytosis motif of CD4 (Garron et al., 2008), and the interaction between AKAP-*IS* and the RIIa subunit of PKA (Gold et al., 2006). In the system studied here, bidirectionality is related to the pseudo-palindromic character of the LD consensus and the resulting resemblance of the interfaces formed by oppositely aligned LD motifs with α-parvin-CH_C_.

Our global analysis of the PRE effects of spin-labeled LD1 (paxillin residues 1–20) on α-parvin-CH_C_ resonances indicates that forward and backward binding modes may be simultaneously accessible to the same peptide in solution, implying similar binding energies for the two modes; the 3:1 apparent ratio observed for LD1 at room temperature corresponds to a difference in binding energy of just 2.7 kJ mol^−1^. Small changes in a variety of factors, such as experimental conditions or the length of the paxillin fragment studied, might influence the observed binding orientation in NMR, where a mean is detected. This could explain a similar PRE experiment performed with a shorter LD1 variant (residues 3–13), which was interpreted as unidirectional forward-type binding and used to restrain the solution structure of the LD1/α-parvin-CH_C_ complex (Wang et al., 2008).

Although our PRE experiments suggest bidirectional binding in solution, the refinement of different models against our X-ray data strongly indicate a predominance of the forward binding mode in the α-parvin-CH_C_/LD1 crystal. At the same time, the TLS-corrected individual isotropic temperature factors of the LD-peptides are not atypical of those expected for weakly bound ligands (data not shown): deviation from such behavior would be expected if the crystal contained significant static disorder in peptide binding. Taken together, these observations suggest that a unidirectional complex is purified to homogeneity by the process of crystallization. In both the forward and backward binding mode, LD peptides make a small number of crystal contacts. The capacity of such relatively weak crystal-packing forces to bring about homogenization of orientation is consistent with our finding that the two different orientations are approximately isoenergetic in solution.

Although α−parvin has been shown to bind to F-actin with an affinity comparable to other CH domain-containing actin-binding proteins (Nikolopoulos and Turner, 2000; Olski et al., 2001), the canonical model of actin recognition may not apply to this protein. Usually CH domains bind F-actin through a tandem array of an N-terminal type-1 CH domain containing two actin-binding sites (ABS1 and ABS2) and a C-terminal type-2 CH domain contributing a third actin-binding site (ABS3) (Gimona et al., 2002). However, both CH domains of α-parvin are atypical and more similar in sequence to type-1 than type-2 CH domains. Despite some sequence conservation in the regions homologous to ABS1 and ABS2, many of the critical residues, which are highly conserved across type-1 CH domains of actin-binding proteins, are absent in α-parvin-CH_C_ (Figure S1). Interestingly, the crystal structure of α−parvin-CH_C_ presented here shows that part of the putative ABS2 region is obstructed by the N-linker helix. We have also shown that helices A and G, which contain the putative ABSs, are involved in the binding of paxillin LD motifs, which could render the interaction of α−parvin with paxillin and F-actin mutually exclusive.

We have shown that LD binding imposes conformational changes in the most N-terminal region of the α-parvin-CH_C_ domain. If these effects are propagated to the N-terminal regions of α-parvin, they might affect the conformation of the inter domain linker and/or potential interactions between the two CH domains. CH domains in intact ABDs often interact across a sizeable interface, which can modulate their ability to interact with F-actin (Gimona et al., 2002). It remains to be established whether such intramolecular interactions occur to regulate the association of α-parvin with F-actin and other binding partners, such as paxillin, TESK1 (LaLonde et al., 2005), and ILK (Tu et al., 2001).

Several ABD containing proteins are capable of forming dimers, thereby cross-linking actin filaments (Gimona et al., 2002). On the basis of gel filtration experiments with the N-terminal CH domain of α-parvin, Wang et al. (2008) speculate that α-parvin might also dimerize. However, the full-length protein is predominantly monomeric under the conditions used in our experiments (data not shown).

Taken together, we have presented a comprehensive structural characterization of the interaction between paxillin LD motifs and α-parvin, which has revealed a surprising degree of promiscuity, both in terms of LD motif selectivity and binding directionality. Although it is unclear how these features are exploited and dynamically regulated in vivo, we speculate that they may have a role in the context of paxillin-mediated supramolecular assemblies. The existence of multiple α-parvin-binding LD motifs in paxillin has at least two potential advantages. Engagement of one motif will increase the effective local concentration of others and render weak interactions functional, even in the presence of stronger LD-binding partners, such as FAK. Two possible binding orientations will also enhance effective association rates in a diffusion-controlled reaction by increasing the number of productive encounters. Such features may allow the assembly of various constitutionally and conformationally distinct protein complexes with specific signaling properties.

## Experimental Procedures

### Proteins

Full-length human α-parvin (1–372) and α-parvin-CH_C_ (242–372) were subcloned from I.M.A.G.E. Consortium cDNA clone no. 4065758 into the expression vector pGEX-6P1 (GE Healthcare) via EcoRI/EcoRI and BamHI/EcoRI restriction sites, respectively. The boundaries of α-parvin-CH_C_ were determined by limited proteolysis: 160 μg α-parvin (residues 223–372) were incubated with 0.1 μg subtilisin A (Sigma) at 25°C for 1 hr; the reaction was stopped by adding protease inhibitor cocktail (Roche), and samples were subjected to N-terminal sequencing and ESI-MS.

Full-length α-parvin and α-parvin-CH_C_ were expressed as GST-fusions in *Escherichia coli* BL21 at 20°C overnight in LB medium. Uniform isotopic enrichment was achieved by using ^15^N-enriched ammonium sulfate (Spectra Stable Isotopes), and unenriched or ^13^C-enriched glucose (Spectra Stable Isotopes). α-parvin-CH_C_ was purified as follows: cleared cell lysate (in 75 mM Tris [pH 8.0], 200 mM NaCl, 5 mM β-mercaptoethanol, 0.4% Triton X-100, 2 mM EDTA, 5 mM benzamidine, and protease inhibitor cocktail [Roche]) was applied to glutathione sepharose 4B (GE Healthcare) in binding buffer (20 mM Tris [pH 8.0], 150 mM NaCl, and 2 mM DTT) washed with 20 mM Tris (pH 8.0), 1 M NaCl, 2 mM DTT, 2 mM EDTA, and 5 mM benzamidine, and was eluted with 50 mM glutathione in binding buffer (pH 8.0). After cleavage with recombinant human rhinovirus 3C-protease, the sample was subjected to size exclusion chromatography (Superdex 75, GE Healthcare) in 25 mM Tris (pH 8.0), 150 mM NaCl, 2 mM DTT, and 2 mM EDTA.

To purify full-length α-parvin, cleared cell lysate in 200 mM potassium phosphate (pH 8.0), 10 mM NaCl, 5 mM β-mercaptoethanol, 0.4% Triton X-100, 5 mM benzamidine, 2 mM EDTA, and protease inhibitor cocktail (Roche) was applied to glutathione sepharose 4B (GE Healthcare), washed with 200 mM potassium phosphate, 10 mM NaCl, and 4 mM DTT, and α−parvin was released by cleavage with recombinant human rhinovirus 3C-protease at 4°C over night. After elution with 200 mM potassium phosphate, 10 mM NaCl, 4 mM DTT, and 2.5% glycerol, the protein was dialyzed into 25 mM potassium phosphate (pH 8.0), 1.5 mM NaCl, 2 mM DTT, and 2.5% (v/v) glycerol for subsequent anion exchange chromatography (MonoQ, GE Healthcare) and gradient elution with 250 mM potassium phosphate (pH 8.0), 15 mM NaCl, 250 mM KCl, 2 mM DTT, and 2.5% glycerol.

### Peptides

All synthetic peptides were purchased from Severn Biotech (Kidderminster, UK) at >95% purity. Concentrations were determined by quantitative amino acid analysis (Alta Bioscience, Birmingham, UK). Peptides representing human paxillin motifs included LD1 (residues 1–20: MDDLDALLADLESTTSHISK), LD2 (141–160: NLSELDRLLLELNAVQHNPP), LD3 (213–232: VRPSVESLLDELESSVPSPV), LD4 (262–281: ATRELDELMASLSDFKFMAQ) and LD5 (296–315: PGSQLDSMLGSLQSDLNKLG). To generate spin-labeled LD1 an N-terminally blocked LD1 peptide in which the N-terminal methionine residue was replaced by cysteine was incubated with a 10-fold molar excess of 3-maleimido-PROXYL (Sigma-Aldrich) at 4°C overnight. After purification by HPLC (Jupiter C18, Phenomonex), quantitative labeling was confirmed by mass spectrometry. For fluorescence anisotropy studies, LD1 with 5-carboxyfluorescein (5-FAM) attached to the ɛ-amino group of the C-terminal lysine was used.

### Crystallization and Data Collection

α-parvin-CH_C_ was crystallized using sitting drop vapor diffusion by mixing 1 μl of protein (7 mg/ml in 25 mM Tris (pH 8.0), 150 mM NaCl, 2 mM DTT, and 2 mM EDTA) with 1 μl of reservoir solution (12% [w/v] PEG 8000, 35% [v/v] MPD, and 0.1 M HEPES [pH 7.5]) at 4°C. The large prism-shaped crystals were flash frozen in mother liquor.

Plate-like α-parvin-CH_C_/LD1 cocrystals were obtained from 8 mg/ml protein in the presence of a 2-fold molar excess of LD1 peptide in 2 μl sitting drops containing buffer 28 (20% [w/v] PEG 10000 and 0.1 M HEPES [pH 7.5]) of Structure Screen 2 (Molecular Dimensions) at 13°C and were flash-frozen in mother liquor including 35% (v/v) glycerol.

Rod-shaped α-parvin-CH_C_/LD2 cocrystals were grown from 9 mg/ml protein in the presence of a 2-fold molar excess of LD2 peptide in sitting drops containing 40% (w/v) PEG 200 and 0.1 M citrate (pH 4.5) at 13°C and were flash-frozen in mother liquor. Rod-shaped α-parvin-CH_C_/LD4 cocrystals were grown under similar conditions (40% [w/v] PEG 300 and 0.1 M citrate [pH 5.2]).

All data were processed using the CCP4 program suite (Collaborative Computational Project, Number 4, 1994).

### Structure Calculation and Refinement

The structure of unliganded α-parvin-CH_C_ was solved by molecular replacement with an ensemble of homologous structures (20%–25% sequence identity) including the CH1 domains of α-actinin1 (residues 30–135 of chain A of 2EYI), α-actinin3 (residues 42–149 of chain B of 1WKU, residues 42–149 of chain A of 1TJT) and plectin (residues 59–172 of chain A of 1MB8) using the program PHASER (McCoy, 2007). Manual model building was performed with COOT (Emsley and Cowtan, 2004), and refinement was performed with REFMAC5 (Murshudov et al., 1997); occupancies of alternate conformers were refined with PHENIX (Afonine et al., 2007).

Structures of α-parvin-CH_C_/LD complexes were solved by molecular replacement with the α-parvin-CH_C_ *apo* structure using PHASER (McCoy, 2007), and refined with REFMAC5, allowing one TLS group per polypeptide chain. In all models, >98% of the residues lie in the favored Ramachandran regions and none in the disallowed regions.

### NMR

Data were recorded on home-built or Bruker spectrometers with 11.7, 14.1, 17.6, and 22.3 T field strengths and processed with NMRPipe (Delaglio et al., 1995). Backbone chemical shift assignments were obtained using standard triple resonance experiments. Peptide titration experiments were performed by mixing two stock solutions (in 50 mM sodium phosphate, 100 mM NaCl, 2 mM DTT, 5% D_2_O, and 30 μM DSS [pH 6.9]) containing 235 μM ^15^N-enriched α-parvin-CH_C_ and either no or a maximum concentration of LD peptide at the required protein/ligand ratios (Figure S2). Phase-sensitive gradient-enhanced ^1^H-^15^N HSQC spectra (Kay et al., 1992) were recorded at 25°C. To compare chemical shift perturbations a weighted combined chemical shift difference Δδ(^1^H^15^N) was calculated according to

Δδ(H1N15)=(δ(H1)sat−δ(H1)0)2+0.04∗(Δδ(N15)sat−δ(H1)0)2.

Binding curves for individual resonances were fitted globally to a single-site model given by

Δδ(H1N15)=Δδ(H1N15)sat([Ptot]+[Ltot]+KD)±([Ptot]+[Ltot]+KD)2−4[Ptot][Ltot]2[Ptot],

where [P_tot_] and [L_tot_] denote the concentrations of α-parvin-CH_C_ and LD peptides, respectively. None of the individual isotherms (with Δδ(^1^H^15^N)_sat_ > 0.2 ppm) showed significant deviations from this model (Figure S2).

For PRE measurements, ^1^H-^15^N HSQC experiments were recorded on 230 μM ^15^N-enriched α-parvin-CH_C_ in the presence of 250 μM PROXYL-labeled LD1 peptide in 50 mM sodium phosphate and 100 mM NaCl (pH 6.9) in the absence and presence of 5 mM ascorbate at 25°C. The intensity ratios I/I_0_ (where I and I_0_ denote peak heights in the absence and presence of ascorbate, respectively) were determined. For the simulation of PRE effects in both unidirectional binding modes, the crystal structures of α-parvin-CH_C_ bound to LD1 and LD4 were protonated with the program PDB 2PQR (Dolinsky et al., 2004). Equivalent peptide residues corresponding to M-3 in LD1 and T-3 in LD4 of the structural models were replaced by cysteine residues and the distances of their S^γ^-atoms to individual backbone amide protons of α-parvin-CH_C_ were determined. The procedure was repeated for 4 different cysteine rotamers and the distance data were averaged. The resulting values were used as approximation of the distances, r, of the unpaired electron of the PROXYL moiety to the backbone amide protons and to derive residue-specific PRE-values, I/I_0_, according to (Jain et al., 2001; Johnson et al., 1999) (for details, see Figure S5).

### Fluorescence Anisotropy

Titrations were performed by mixing two stock solutions containing 50 nM 5-FAM-labeled LD1 peptide and either no or 300 μM protein in 100 mM potassium phosphate and 2 mM DTT (pH 8.0). Measurements on a SpectraMax M5 Microplate Reader (Molecular Devices) at 25°C with excitation and emission wavelengths of 485 and 538 nm, respectively, were repeated four times, and data were fitted globally to a single-site model (see above).

## Figures and Tables

**Table 1:** Data Collection and Refinement Statistics

Data Collection				
Name	CH_C_	CH_C_/LD1	CH_C_/LD2	CH_C_/LD4
Space group	P2_1_	C2	C222_1_	C222_1_
Cell dimensions	44.14, 71.20, 47.15	133.12, 37.50, 70.24	74.01, 94.42, 41.84	75.42, 94.60, 42.17
a,b,c (Å)	90.0, 99.88, 90.00	90.00, 90.33, 90.00	90.00, 90.00, 90.00	90.00, 90.00, 90.00
α,β,γ (°)				
X-ray source	ESRF ID14-2	ESRF ID23-1	DLS-I04	ESRF-ID29
Wavelength (Å)	0.933	0.969	0.968	0.976
Resolution (Å)	23.74−1.05 (1.11−1.05)	28.64−2.10 (2.21−2.10)	27.71−1.80 (1.90−1.80)	37.72−2.20 (2.32−2.20)
I/σI	16.5 (2.4)	16.4 (2.5)	13.2 (3.2)	9.7 (2.2)
Completeness (%)	93.7∗ (77.7)	98.5 (99.4)	99.6 (100)	99.5 (100)
R_sym_ (%)	4.0 (46.0)	5.4 (44.9)	7.1 (35.1)	11.9 (63.4)
Redundancy	4.2 (3.5)	2.9 (3.0)	3.4 (3.5)	3.5 (3.6)
Model Refinement				
Resolution (Å)	1.05	2.1	1.8	2.2
R_work_/R_free_ (%)	14.3/15.9	21.7/25.5	18.6/22.1	20.5/26.0
No. atoms				
Protein(+ peptide)	2777	2765	1373	1333
Ligand/ion	30	92	43	41
Water	279	85	65	39
B-factor average				
Protein	11.5	42.2	24.3	30.90
Peptide		44.7	27.7	35.08
RMS deviations				
Bond lengths (Å)	0.014	0.007	0.010	0.008
Bond angles (°)	1.69	1.13	1.23	1.14

Values in parentheses are for the highest-resolution shell. One crystal was used for each data collection.

∗ >96% in each shell until 1.3 Å resolution.

**Table 2:** Dissociation Constants for the Interaction of α-Parvin with Paxillin LD Motifs

Protein	Peptide	K_D_ (μM)	Method
α-parvin-CH_C_	LD1	96 ± 2	NMR
α-parvin-CH_C_	LD2	204 ± 3	NMR
α-parvin-CH_C_	LD3	2300 ± 100	NMR
α-parvin-CH_C_	LD4	140 ± 30	NMR
α-parvin-CH_C_	LD5	mM range^a^	NMR
α-parvin-CH_C_	LD1	120 ± 20	Fluorescence anisotropy
full-length α-parvin	LD1	130 ± 22	Fluorescence anisotropy

a No accurate K_D_-value could be obtained for LD5 due to its low affinity for α−parvin-CH_C_.

## Accession Codes

Atomic coordinates and structure factors have been deposited in the Protein Data Bank with accession codes 2VZC, 2VZD, 2VZG, and 2VZI for α-parvin-CH_C_, CH_C_/LD1, CH_C_/LD2, and CH_C_/LD4, respectively. α-parvin-CH_C_ backbone resonance assignments have been deposited in the BioMagResBank under accession number 15899.

## Supplemental Data

Document S1. Six Figures and Supplemental References

## References

[bib1] Afonine P.V., Grosse-Kunstleve R.W., Adams P.D., Lunin V.Y., Urzhumtsev A. (2007). On macromolecular refinement at subatomic resolution with interatomic scatterers. Acta Crystallogr. D Biol. Crystallogr..

[bib2] Ball L.J., Kuhne R., Schneider-Mergener J., Oschkinat H. (2005). Recognition of proline-rich motifs by protein-protein-interaction domains. Angew. Chem. Int. Ed. Engl..

[bib3] Brown M.C., Perrotta J.A., Turner C.E. (1996). Identification of LIM3 as the principal determinant of paxillin focal adhesion localization and characterization of a novel motif on paxillin directing vinculin and focal adhesion kinase binding. J. Cell Biol..

[bib4] Brown M.C., Turner C.E. (2004). Paxillin: adapting to change. Physiol. Rev..

[bib5] Brubaker K., Cowley S.M., Huang K., Loo L., Yochum G.S., Ayer D.E., Eisenman R.N., Radhakrishnan I. (2000). Solution structure of the interacting domains of the Mad-Sin3 complex: implications for recruitment of a chromatin-modifying complex. Cell.

[bib6] Delaglio F., Grzesiek S., Vuister G.W., Zhu G., Pfeifer J., Bax A. (1995). NMRPipe: a multidimensional spectral processing system based on UNIX pipes. J. Biomol. NMR.

[bib7] Djinovic Carugo K., Banuelos S., Saraste M. (1997). Crystal structure of a calponin homology domain. Nat. Struct. Biol..

[bib8] Dolinsky T.J., Nielsen J.E., McCammon J.A., Baker N.A. (2004). PDB2PQR: an automated pipeline for the setup of Poisson-Boltzmann electrostatics calculations. Nucleic Acids Res..

[bib9] Emsley P., Cowtan K. (2004). Coot: model-building tools for molecular graphics. Acta Crystallogr. D Biol. Crystallogr..

[bib10] Fukuda T., Guo L., Shi X., Wu C. (2003). CH-ILKBP regulates cell survival by facilitating the membrane translocation of protein kinase B/Akt. J. Cell Biol..

[bib11] Gao G., Prutzman K.C., King M.L., Scheswohl D.M., DeRose E.F., London R.E., Schaller M.D., Campbell S.L. (2004). NMR solution structure of the focal adhesion targeting domain of focal adhesion kinase in complex with a paxillin LD peptide: evidence for a two-site binding model. J. Biol. Chem..

[bib12] Garron M.L., Arthos J., Guichou J.F., McNally J., Cicala C., Arold S.T. (2008). Structural basis for the interaction between focal adhesion kinase and CD4. J. Mol. Biol..

[bib13] Gimona M., Djinovic-Carugo K., Kranewitter W.J., Winder S.J. (2002). Functional plasticity of CH domains. FEBS Lett..

[bib14] Gold M.G., Lygren B., Dokurno P., Hoshi N., McConnachie G., Tasken K., Carlson C.R., Scott J.D., Barford D. (2006). Molecular basis of AKAP specificity for PKA regulatory subunits. Mol. Cell.

[bib15] Hashimoto S., Tsubouchi A., Mazaki Y., Sabe H. (2001). Interaction of paxillin with p21-activated Kinase (PAK). Association of paxillin alpha with the kinase-inactive and the Cdc42-activated forms of PAK3. J. Biol. Chem..

[bib16] Hoellerer M.K., Noble M.E., Labesse G., Campbell I.D., Werner J.M., Arold S.T. (2003). Molecular recognition of paxillin LD motifs by the focal adhesion targeting domain. Structure.

[bib17] Jain N.U., Venot A., Umemoto K., Leffler H., Prestegard J.H. (2001). Distance mapping of protein-binding sites using spin-labeled oligosaccharide ligands. Protein Sci..

[bib18] Johnson P.E., Brun E., MacKenzie L.F., Withers S.G., McIntosh L.P. (1999). The cellulose-binding domains from Cellulomonas fimi beta−1, 4-glucanase CenC bind nitroxide spin-labeled cellooligosaccharides in multiple orientations. J. Mol. Biol..

[bib19] Kay L., Keifer P., Saarinen T. (1992). Pure absorption gradient enhanced heteronuclear single quantum correlation spectroscopy with improved sensitivity. J. Am. Chem. Soc..

[bib20] Krissinel E., Henrick K. (2004). Secondary-structure matching (SSM), a new tool for fast protein structure alignment in three dimensions. Acta Crystallogr. D Biol. Crystallogr..

[bib21] LaLonde D.P., Brown M.C., Bouverat B.P., Turner C.E. (2005). Actopaxin interacts with TESK1 to regulate cell spreading on fibronectin. J. Biol. Chem..

[bib22] Liu G., Guibao C.D., Zheng J. (2002). Structural insight into the mechanisms of targeting and signaling of focal adhesion kinase. Mol. Cell. Biol..

[bib23] McCoy A.J. (2007). Solving structures of protein complexes by molecular replacement with Phaser. Acta Crystallogr. D Biol. Crystallogr..

[bib24] Murshudov G.N., Vagin A.A., Dodson E.J. (1997). Refinement of macromolecular structures by the maximum-likelihood method. Acta Crystallogr D Biol. Crystallogr..

[bib25] Nikolopoulos S.N., Turner C.E. (2000). Actopaxin, a new focal adhesion protein that binds paxillin LD motifs and actin and regulates cell adhesion. J. Cell Biol..

[bib26] Nikolopoulos S.N., Turner C.E. (2001). Integrin-linked kinase (ILK) binding to paxillin LD1 motif regulates ILK localization to focal adhesions. J. Biol. Chem..

[bib27] Olski T.M., Noegel A.A., Korenbaum E. (2001). Parvin, a 42 kDa focal adhesion protein, related to the alpha−actinin superfamily. J. Cell Sci..

[bib28] Scheswohl D.M., Harrell J.R., Rajfur Z., Gao G., Campbell S.L., Schaller M.D. (2008). Multiple paxillin binding sites regulate FAK function. J Mol. Signal..

[bib29] Schmalzigaug R., Garron M.L., Roseman J.T., Xing Y., Davidson C.E., Arold S.T., Premont R.T. (2007). GIT1 utilizes a focal adhesion targeting-homology domain to bind paxillin. Cell. Signal..

[bib30] Schultz J., Milpetz F., Bork P., Ponting C.P. (1998). SMART, a simple modular architecture research tool: identification of signaling domains. Proc. Natl. Acad. Sci. USA.

[bib31] Sheibani N., Tang Y., Sorenson C.M. (2008). Paxillin's LD4 motif interacts with bcl-2. J. Cell. Physiol..

[bib32] Sorenson C.M. (2004). Interaction of bcl-2 with Paxillin through its BH4 domain is important during ureteric bud branching. J. Biol. Chem..

[bib33] Spronk C.A., Tessari M., Kaan A.M., Jansen J.F., Vermeulen M., Stunnenberg H.G., Vuister G.W. (2000). The Mad1-Sin3B interaction involves a novel helical fold. Nat. Struct. Biol..

[bib34] Swanson K.A., Knoepfler P.S., Huang K., Kang R.S., Cowley S.M., Laherty C.D., Eisenman R.N., Radhakrishnan I. (2004). HBP1 and Mad1 repressors bind the Sin3 corepressor PAH2 domain with opposite helical orientations. Nat. Struct. Mol. Biol..

[bib35] Thomas J.W., Cooley M.A., Broome J.M., Salgia R., Griffin J.D., Lombardo C.R., Schaller M.D. (1999). The role of focal adhesion kinase binding in the regulation of tyrosine phosphorylation of paxillin. J. Biol. Chem..

[bib36] Tong X., Salgia R., Li J.L., Griffin J.D., Howley P.M. (1997). The bovine papillomavirus E6 protein binds to the LD motif repeats of paxillin and blocks its interaction with vinculin and the focal adhesion kinase. J. Biol. Chem..

[bib37] Tu Y., Huang Y., Zhang Y., Hua Y., Wu C. (2001). A new focal adhesion protein that interacts with integrin-linked kinase and regulates cell adhesion and spreading. J. Cell Biol..

[bib38] Turner C.E., Brown M.C., Perrotta J.A., Riedy M.C., Nikolopoulos S.N., McDonald A.R., Bagrodia S., Thomas S., Leventhal P.S. (1999). Paxillin LD4 motif binds PAK and PIX through a novel 95-kD ankyrin repeat, ARF-GAP protein: A role in cytoskeletal remodeling. J. Cell Biol..

[bib39] Wang X., Fukuda K., Byeon I.-J., Velyvis A., Wu C., Gronenborn A., Qin J. (2008). The structure of α-parvin CH2/paxillin LD1 complex reveals a novel modular recognition for focal adhesion assembly. J. Biol. Chem..

[bib40] Yamaji S., Suzuki A., Kanamori H., Mishima W., Yoshimi R., Takasaki H., Takabayashi M., Fujimaki K., Fujisawa S., Ohno S., Ishigatsubo Y. (2004). Affixin interacts with alpha−actinin and mediates integrin signaling for reorganization of F-actin induced by initial cell-substrate interaction. J. Cell Biol..

[bib41] Yoshimi R., Yamaji S., Suzuki A., Mishima W., Okamura M., Obana T., Matsuda C., Miwa Y., Ohno S., Ishigatsubo Y. (2006). The gamma-parvin-integrin-linked kinase complex is critically involved in leukocyte-substrate interaction. J. Immunol..

[bib42] Zaidel-Bar R., Itzkovitz S., Ma'ayan A., Iyengar R., Geiger B. (2007). Functional atlas of the integrin adhesome. Nat. Cell Biol..

[bib43] Zhang Y., Chen K., Tu Y., Wu C. (2004). Distinct roles of two structurally closely related focal adhesion proteins, alpha-parvins and beta-parvins, in regulation of cell morphology and survival. J. Biol. Chem..

[bib44] Zhang Z.M., Simmermann J.A., Guibao C.D., Zheng J.J. (2008). GIT1 paxillin-binding domain is a four-helix bundle and it binds to both paxillin LD2 and LD4 motifs. J. Biol. Chem..

