# Supplementary material for: Structural Analysis of the Interactions Between Paxillin LD Motifs and α-Parvin
Source: Structure. 2008 Oct 8;16(10-24):1521–31. doi: 10.1016/j.str.2008.08.007 (PMC2572193; doi:10.1016/j.str.2008.08.007)
Supplement: Document S1. Six Figures and Supplemental References [file mmc1.pdf]

Structure *16*

Supplemental Data

Structural Analysis of the Interactions Between Paxillin LD Motifs and α-Parvin

Sonja Lorenz, Ioannis Vakonakis, Edward D. Lowe, Iain D. Campbell, Martin E. M. Noble, and Maria K. Hoellerer

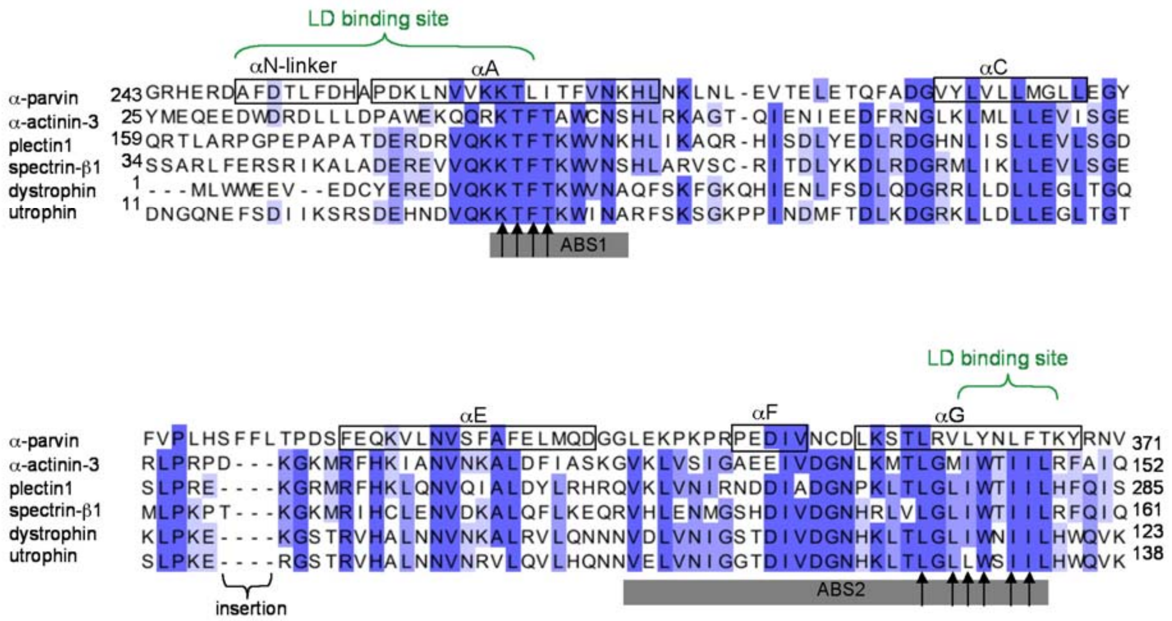

Figure S1. Sequence Alignment of α–Parvin-CH_C with Homologous Type-1 CH domains
Secondary structural elements and the characteristic C/E-loop insertion and N-linker helix of α-parvin are indicated. Regions forming the LD binding site are labelled in green. Grey boxes denote the actin binding sites ABS1 and ABS2, which are conserved across type-1 CH domains; black arrows highlight residues within ABS1 (Hemmings, 1992) and ABS2 (Kuhlman, 1992) that are critical for the actin binding activity of α-actinin.

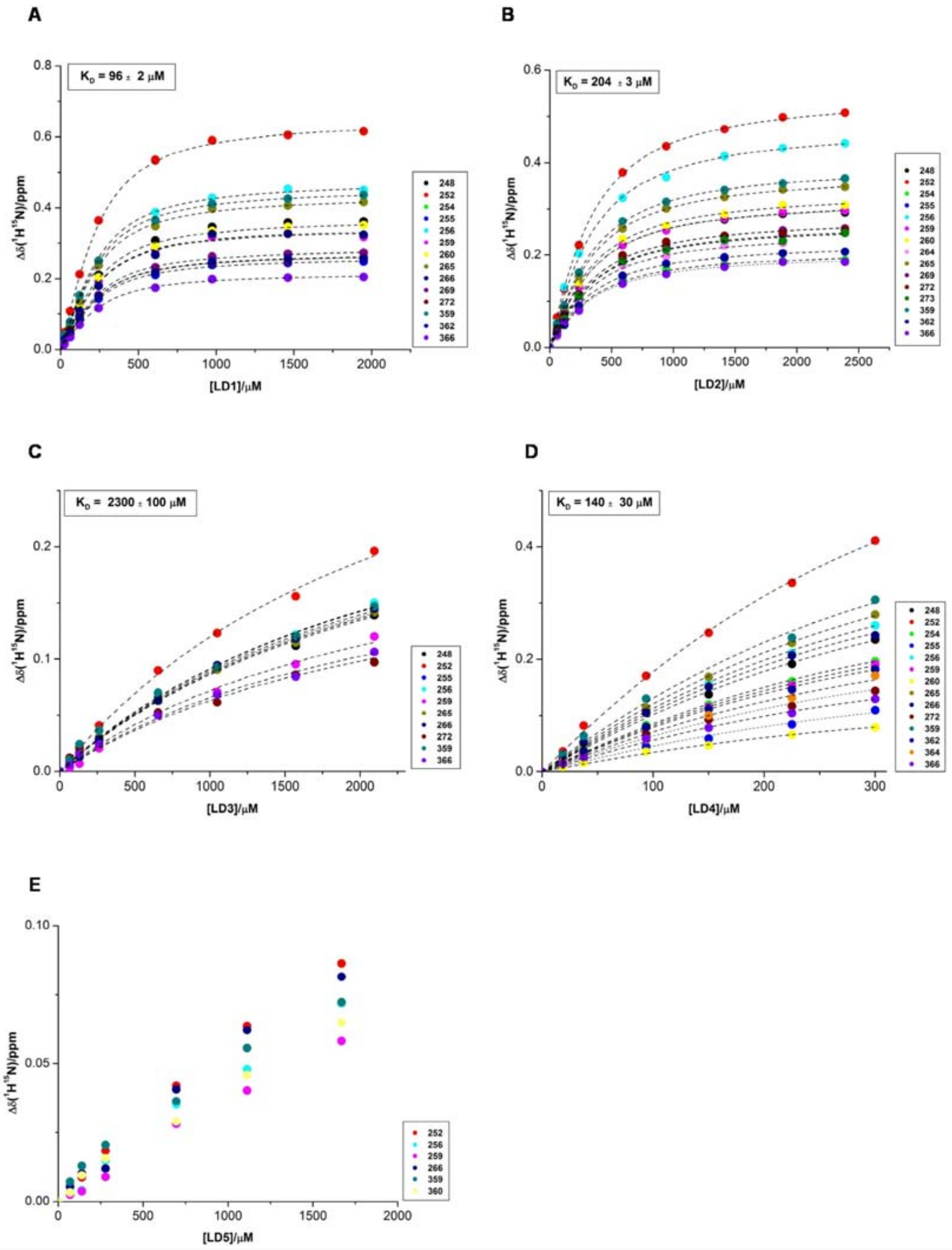

Figure S2. Determination of K_D-Values for the Interaction of α–Parvin-CH_C with LD Peptides by NMR

Titrations were performed with peptides representing the LD1 (A), LD 2(B), LD3 (C), LD4 (D), and LD5 (E) motifs and the corresponding binding curves for significantly perturbed resonances (i.e. $\delta\Delta(^1H^{15}N) > 0.2$ ppm for LD1 and LD2) were fitted globally to a single-site model (see experimental procedures). The errors refer to the fit only and exclude experimental errors. Note that errors are higher for LD3 and LD4, due to relatively low affinity and poor solubility, respectively. No value could be derived for LD5 due to its low affinity. The K_D-value for LD1 represents the weighted average over both antiparallel binding orientations, which exchange fast on the NMR timescale.

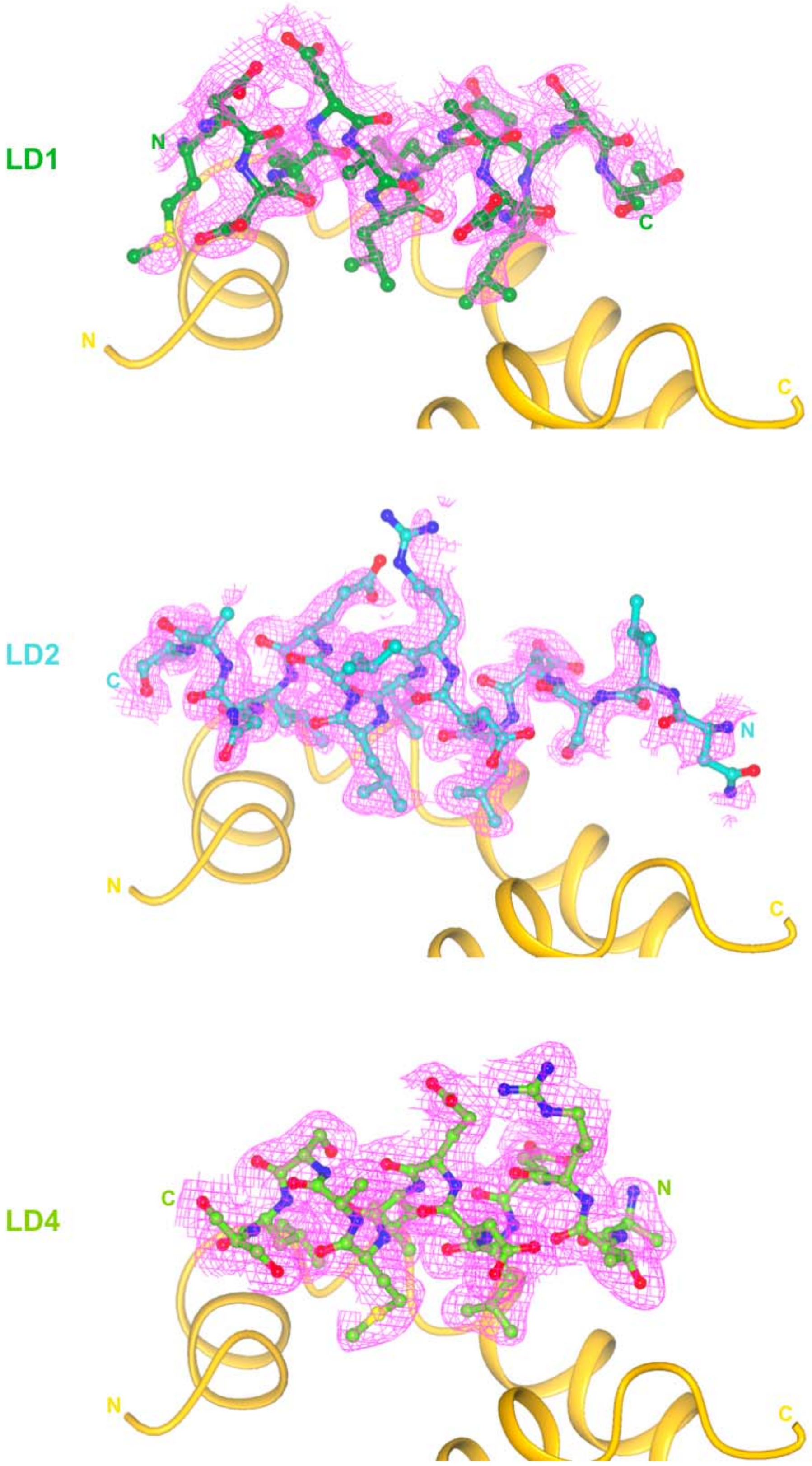

Figure S3. The Position and Orientation of LD Peptides with Respect to α–Parvin-CH$_C$ Is Well-Defined in the Corresponding Co-Crystal Structures
Detail of the α–parvin-CH$_C$ co-crystal structures with LD1 (top), LD2 (middle) and LD4 (bottom). α–parvin-CH$_C$ is shown as ribbon representation; the peptides are displayed in ball-and-stick mode with the respective electron density omit maps contoured at 1σ.

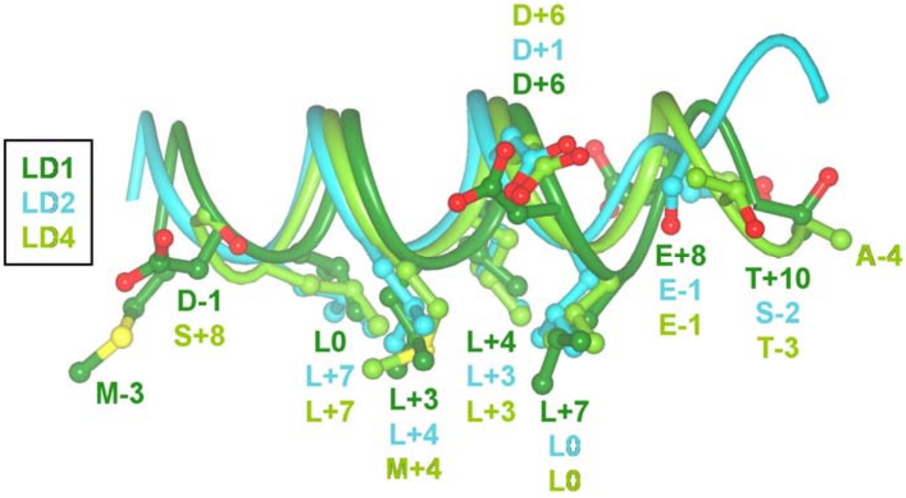

Figure S4. The Position and Character of Peptide Side Chains Across Different α-Parvin-LD Complexes Is Conserved Irrespective of the Binding Orientation

Superposition of the ribbon representations of LD1 (green), LD2 (cyan) and LD4 (light green) in complex with α–parvin-CH_C (not shown). Side-chains of residues within a radius of 4 Å of the protein are shown as ball-and-stick models.

A

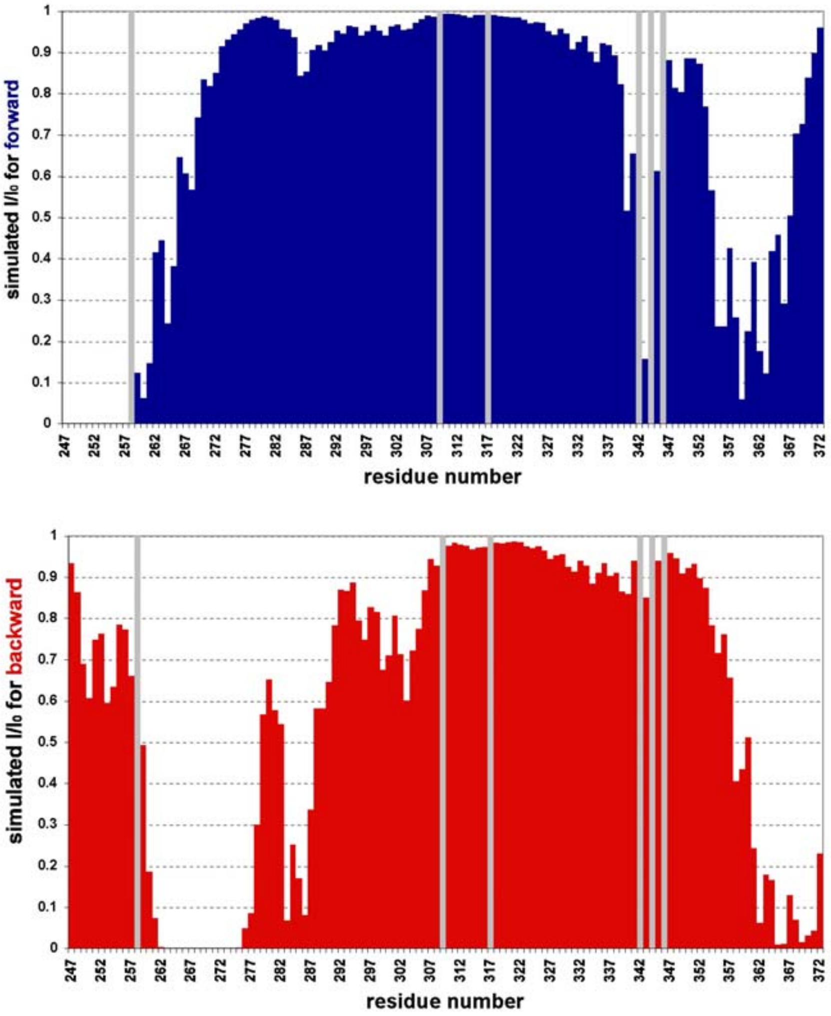

B

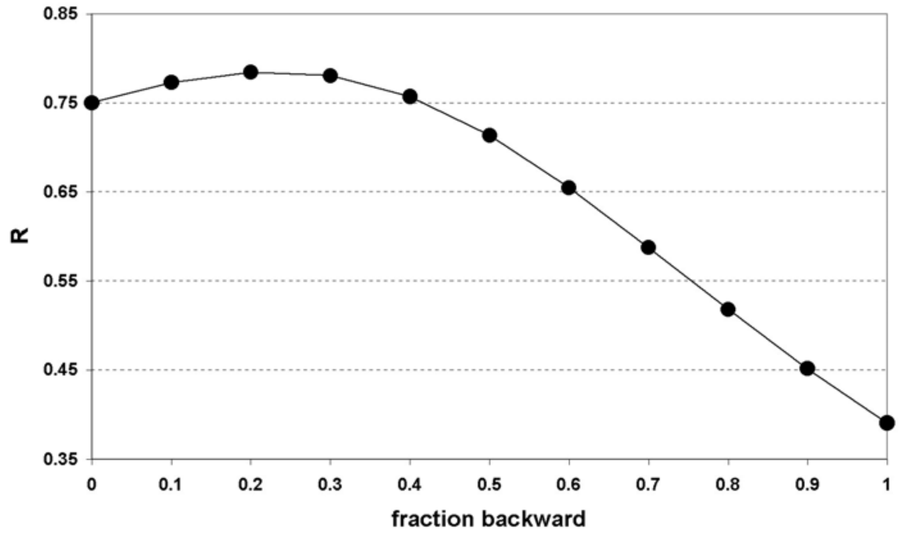

Figure S5. Global PRE Analysis

(A) Comparison of the experimental I/I_0-values (green) and simulated values for pure forward (blue) and backward (red) binding of LD1 peptide to α–parvin-CH_C, respectively. I denotes the intensity of resonances in the presence of spin-labelled LD1 peptide, I_0 the intensity upon addition of 5 mM ascorbate to the protein-peptide mixture. Grey bars indicate the position of prolines, unassigned or overlapped resonances. For the simulation of PRE effects in both unidirectional binding modes, the distances, r, of the unpaired electron of the PROXYL moiety to the backbone amide protons were estimated on the basis of the crystal structures of α–parvin-CH_C bound to LD1 and LD4, respectively, as described in the text. Note that the structure of LD4 was used here rather than LD2, since the N-terminus of the latter deviates from α-helicity, possibly due to crystal packing (Figure 3A). Subsequently, residue-specific PRE-values, I/I_0, were derived according to (Jain et al., 2001; Johnson et al., 1999)

$$\frac{I}{I_0} = \left(\exp\left(t\left(\left(\frac{1}{T_2}\right) - \left(\frac{1}{T_2}\right)_0\right)\right)\right)^{-1} = \exp\left(\frac{tK}{r^6}\left(4\tau_c + \frac{3\tau_c}{1+\omega^2\tau_c^2}\right)\right)^{-1}$$

where $(1/T_2)$ and $(1/T_2)_0$ denote the transverse relaxation rates in the presence and absence of spin label. The parameter t denotes the total time of the INEPT (insensitive nuclei enhanced by polarization transfer) and reverse-INEPT periods of the HSQC pulse sequence, during which the amide proton magnetization experiences paramagnetic relaxation, in this case 10.6 ms. K is given by $1/15*S(S+1)g^2g^2b^2 = 1.23*10^{-32}\ cm^6s^{-2}$) (Jain *et al.*, 2001) where S denotes the electron spin, γ the proton gyromagnetic ratio, g the electronic g-factor, b the Bohr magneton, r the distance between the electron spin and the nuclear spin. t_c, the rotational correlation time of the vector connecting the electron and nuclear spins was estimated to be 8.5 ns based on literature values (Su et al., 2007) and ω, the ^1H Larmor frequency, was 950 MHz. I and I_0 were determined as peak heights rather than volumes, since they could be determined more accurately and for a larger number of resonances.

Note that the experimental I/I_0 profile resembles a mixture of the two simulated unidirectional profiles.

(B) Plot of the linear correlation coefficient, R, between the experimental and simulated I/I_0-values for various fractions of the backward orientation in a bidirectional binding event. The best correlation is obtained for a combination of ~ 75% forward and ~ 25% backward binding.

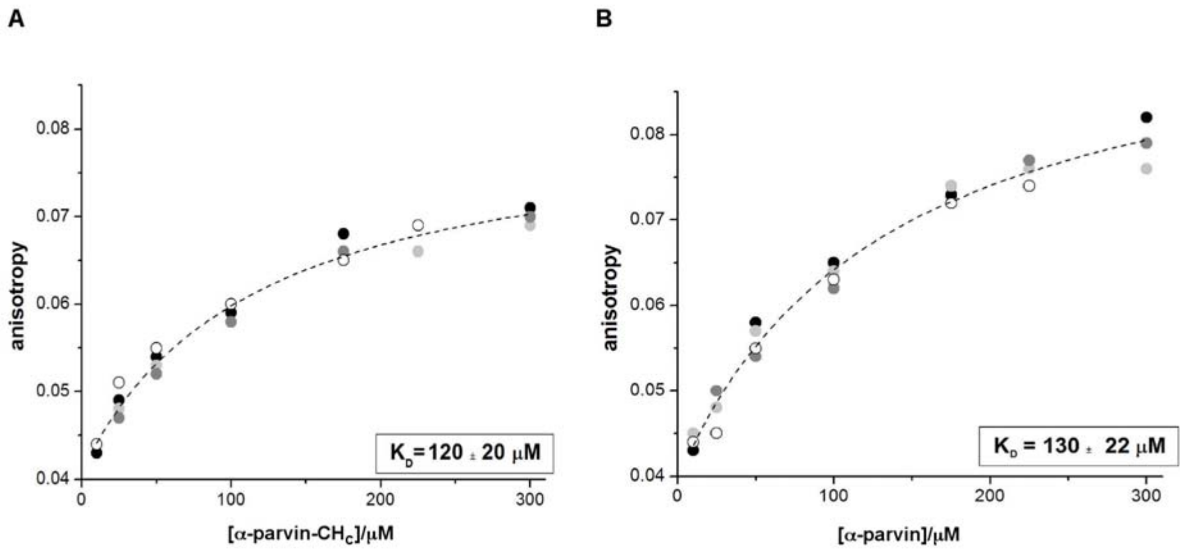

Figure S6. Determination of K_D-Values for the Interaction of the LD1 Motif with α–Parvin-CH_C and Full-Length α–Parvin, Respectively, by Fluorescence Anisotropy
Four replicate datasets for the interaction of C-terminally 5FAM-labelled LD1 peptide with α–parvin-CH_C (A) and full-length α–parvin (B) were fitted globally to a single site model (dashed line).

Supplemental References

Hemmings, L., Kuhlman, P.A., and Critchley, D.R. (1992). Analysis of the actin-binding domain of alphα–actinin by mutagenesis and demonstration that dystrophin contains a functionally homologous domain. J. Cell Biol. *116*, 1369-1380.

Jain, N.U., Venot, A., Umemoto, K., Leffler, H., and Prestegard, J.H. (2001). Distance mapping of protein-binding sites using spin-labeled oligosaccharide ligands. Protein Sci. *10*, 2393-2400.

Johnson, P.E., Brun, E., MacKenzie, L.F., Withers, S.G., and McIntosh, L.P. (1999). The cellulose-binding domains from Cellulomonas fimi betα–1, 4-glucanase CenC bind nitroxide spin-labeled cellooligosaccharides in multiple orientations. J. Mol. Biol. *287*, 609-625.

Kuhlman, P.A., Hemmings, L., and Critchley, D.R. (1992). The identification and characterisation of an actin-binding site in alphα–actinin by mutagenesis. FEBS Lett. *304*, 201-206.

Su, X.C., Jergic, S., Ozawa, K., Burns, N.D., Dixon, N.E., and Otting, G. (2007). Measurement of dissociation constants of high-molecular weight protein-protein complexes by transferred ^{15}N-relaxation. J. Biolmol. NMR *38*, 65-72.
